# The Fate of Contaminants of Emerging Concern in an Upflow Anaerobic Sludge Blanket Reactor Coupled with Constructed Wetlands for Decentralized Domestic Wastewater Treatment

**DOI:** 10.3390/molecules30132671

**Published:** 2025-06-20

**Authors:** Evridiki Barka, Asimina Koukoura, Evangelos Statiris, Taxiarchis Seintos, Athanasios S. Stasinakis, Daniel Mamais, Simos Malamis, Constantinos Noutsopoulos

**Affiliations:** 1 Sanitary Engineering Laboratory, Department of Water Resources and Environmental Engineering, School of Civil Engineering, National Technical University of Athens, Zographou Campus, 9 Iroon Polytechniou St., 15773 Athens, Greece; ebarka8@gmail.com (E.B.); vagstatiris@gmail.com (E.S.); sei_taxiarchis@hotmail.com (T.S.); mamais@central.ntua.gr (D.M.); malamis.simos@gmail.com (S.M.); 2 Water and Air Quality Laboratory, Department of Environment, University of the Aegean, University Hill, 81100 Mytilene, Greece; akoukoura@env.aegean.gr (A.K.); astas@env.aegean.gr (A.S.S.)

**Keywords:** non-steroidal anti-inflammatory drugs, endocrine disrupting chemicals, benzotriazoles, benzothiazoles, decentralized treatment, upflow anaerobic sludge blanket reactor, constructed wetlands, biodegradation, sorption, pilot scale

## Abstract

Removal of micropollutants using biological treatment systems remains a challenge, since conventional bioprocess systems require adaptations to provide more advanced treatment. An ambient temperature upflow anaerobic sludge blanket (UASB) reactor was employed, followed by a two-stage (saturated and unsaturated) vertical subsurface flow (VSSF) constructed wetland (CW) system, to treat domestic wastewater from a nearby settlement and investigate the occurrence and fate of 10 contaminants of emerging concern (CECs) in decentralized, non-conventional treatment systems. The integrated UASB—two-stage CW system achieved high performance regarding abatement of target CECs across all periods. Removal efficiencies ranged from 78% ± 21% (ketoprofen) to practically 100% (2-hydroxybenzothiazole). The pilot system was found to be robust performance-wise and provided enhanced treatment in comparison to a conventional wastewater treatment plant operating in parallel. Most of the target CECs were successfully treated by UASB, saturated and unsaturated CWs, while ibuprofen, bisphenol A and diclofenac were mostly removed in the unsaturated CW. Environmental risk assessment revealed that triclosan poses a significant ecological risk to algae during treated wastewater disposal into the aquatic environment. Additionally, cumulative risk quotient indicated that the potential for mixture toxicity should be carefully considered across all trophic levels.

## 1. Introduction

Organic micropollutants leach into the water as a result of various industrial, rural, and domestic uses and are recognized as a threat to the environment and human health. In fact, water-related legislation is being updated to tackle their presence in water sources and the related risk of leaving water unsafe and toxic for human consumption. A recent example is the revised European Urban Wastewater Treatment Directive 2024/3019 (https://eur-lex.europa.eu/eli/dir/2024/3019/oj, accessed on 11 April 2025) that sets an obligation for the removal of selected micropollutants [1].

Removal of micropollutants using biological treatment systems remains a challenge, since conventional bioprocess systems, e.g., conventional activated sludge (CAS), cannot effectively eliminate organic micropollutants, while performance of alternative biological processes should be evaluated [1]. In the Mediterranean region, anaerobic reactors have been increasingly utilized for domestic wastewater treatment due to their efficiency and suitability to the local climate [2]. In general, anaerobic reactors, including anaerobic baffled reactors (ABRs), anaerobic membrane bioreactors (AnMBRs), anaerobic moving bed biofilm reactors (AnMBBRs) and upflow anaerobic sludge blanket (UASB) reactors are employed in wastewater treatment to degrade organic pollutants, having shown varying effectiveness in removing contaminants of emerging concern (CECs) [3]. ABRs, for example, have demonstrated the ability to degrade certain CECs, such as perchlorate, nitrophenols, and antibiotics, without adversely affecting chemical oxygen demand (COD) removal efficiency. However, for more complex organic CECs, an additional aerobic treatment step may be necessary to promote oxidation [4]. AnMBRs combine anaerobic metabolism with membrane filtration, enhancing performance stability and microbial diversity, which can improve biodegradation of various CECs compared to traditional anaerobic digestion systems [5]. UASB reactors and AnMBBRs were only able to achieve substantial removals for a few compounds, while others were removed only partially or to low extent [6]. Considering these findings, the removal efficiency of CECs in anaerobic reactors can be inconsistent and the formation of transformation products during treatment requires further investigation to fully comprehend the fate of these contaminants in such systems [7]. Additionally, the coupling of anaerobic reactors with alternative aerobic biological treatment processes should be evaluated in order to achieve efficient micropollutant removal.

Constructed wetlands (CWs) are engineered systems that mimic natural wetlands to treat domestic wastewater effectively, removing conventional pollutants such as organic matter and nutrients [8]. CWs are recognized as an effective and inexpensive technology for wastewater treatment and the effluent quality of the systems has greater potential for water reuse, as the requirements set by the European Regulation 2020/741 [9] can be met. In this aspect, due to their low investment and operation cost combined with good effluent quality and high public acceptance, CWs are proposed as an environmentally friendly technology for the removal of CECs [10]. Conflicting results are reported in the literature regarding the effectiveness of CWs in removing micropollutants compared to conventional treatment processes, such as CAS technology [11]. On the other hand, the effectiveness of CWs in removing endocrine-disrupting chemicals (EDCs) has been demonstrated to be as good as or even better than that of conventional wastewater treatment processes [12]. The removal of CECs in CWs is facilitated through a combination of physical, chemical, and biological processes. Key mechanisms include sorption into substrates, microbial biodegradation, photodegradation, and phytoremediation by wetland vegetation [13]. Avila et al. [14] investigated a hybrid CW, and suggested that high removal efficiency was the result of a combination of abiotic/biotic removal pathways. It was proposed that most organic matter removal, as well as a major part of CEC removal, took place in a vertical flow (VF) CW, where aerobic conditions—induced by an unsaturated bed and intermittent feeding—prevailed. It was also found that, since significant denitrification occurred at anoxic sites or in micropores at the bottom layers of the CW bed, alternative CEC removal processes based on anaerobic microbial metabolism could simultaneously occur and contribute, to a lower extent. In the case of horizontal flow (HF) CWs, in which anaerobic metabolism and sorption into the gravel bed are anticipated as prevailing mechanisms, reduced CEC removal is to be expected, while photodegradation seems to be an important factor in free water surface (FWS) CWs [14].

So far, there is limited information on the effectiveness of UASB–CW systems to remove organic micropollutants. In a previous study [15], the removal efficiency of 16 pharmaceuticals and personal care products (PPCPs) from urban wastewater was assessed in a hybrid pilot plant consisting of a UASB reactor followed by two sequentially connected HF CWs: a surface flow (SF) CW and a subsurface flow wetland (SSF) CW. While PPCPs removal associated with the dissolved phase exhibited a seasonal pattern, the fraction linked to suspended solids showed less seasonal variation. The efficiency of the different treatment steps was also found to be compound-dependent, but the SF CW generally exhibited the highest removal efficiency for most of the contaminants analyzed. A recent review [16] concluded that the CWs’ performance was similar to other wastewater treatment plants (WWTPs), while anaerobic reactors (AR) demonstrated almost half the efficiency in CEC removal in comparison with CWs. It was further proposed that the combination of ARs and VF CWs constitute a compact and intensified treatment scheme that incorporates both aerobic and anaerobic conditions, which can enhance the removal of a large group of CECs, and provide high-efficiency removal of organic matter and nitrogen. Sanchez et al. [17] investigated the efficiency of CEC removal of a coupled hybrid anaerobic reactor (UASB at the bottom and anaerobic-aerobic filter on the top)—VF CW system. As it was proposed, the coupled system was able to nearly eliminate certain compounds exclusively during the aerobic stages, while other compounds were removed across all treatment stages. Some CECs exhibiting recalcitrant behavior during biological treatment were not successfully removed.

In the present work, an ambient temperature UASB reactor was employed, followed by a two-stage vertical subsurface flow (VSSF) CW system. Its aim was to treat domestic wastewater from a nearby settlement and investigate the occurrence and fate of 10 CECs in decentralized, non-conventional treatment systems. To the best of the authors’ knowledge, this is the first application where a non-heated anaerobic reactor, followed by two different VF–CW configurations, is evaluated for its capacity to remove CECs and compared to a conventional treatment system (extended aeration CAS WWTP) operating in parallel on the same raw wastewater. The pilot system’s overall efficiency was assessed at three different operational periods to investigate the effect of seasonal variations of temperature, influent CEC concentration, and capacity in terms of daily flowrate. The investigation also assessed the contribution of each treatment step to the overall removal efficiency, considering the prevailing mechanisms and the potential effects of operational parameters at each stage. Finally, environmental risks associated with the disposal of treated wastewater into the aquatic environment was evaluated with regards to three aquatic organisms: fish, *Daphnia magna*, and algae.

## 2. Materials and Methods

### 2.1. Conventional WWTP

In the case of the Antissa, Lesvos Island, Greece, a wastewater treatment plant was in operation prior to the implementation of the pilot system. The WWTP provided secondary treatment, and the treated effluent was discharged in a nearby water body. The system consists of pretreatment with screening and sand and grit removal, an equalization tank, an anoxic tank, an aerobic bioreactor, and a sedimentation tank, as well as a chlorination tank for the treated effluent.

### 2.2. Pilot System

**Figure 1:**
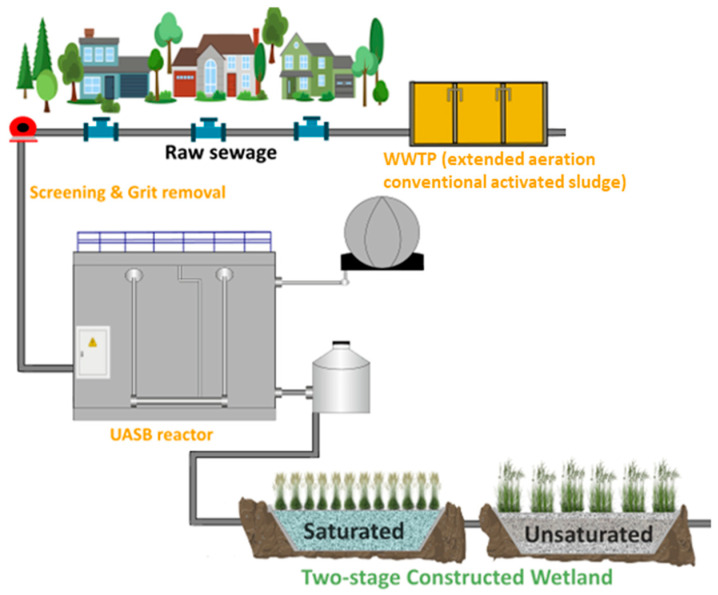
The integrated UASB—VSSF CW system in Antissa, along with the existing activated sludge WWTP.

The novel pilot system (Figure 1) that was investigated towards the removal efficiency of organic micropollutants is a demo site as part of the CARDIMED project (https://www.cardimed-project.eu/, accessed on 8 April 2025) and it was implemented to partially replace the existing extended aeration activated sludge WWTP. The conventional WWTP system is referred to as conv-WWTP hereafter. The pilot system treats domestic wastewater originating from Antissa village, which is on Lesvos Island, Greece. The novel system includes a UASB reactor treating wastewater at ambient temperature coupled with a two-stage vertical subsurface flow constructed wetland (VSSF CW) (Figure 1). Different conditions prevail at the CW of each stage. The first stage is a saturated VSSF CW (VSSF SAT CW) planted with Phragmites Australis and filled with fine gravel that aimed to enhance organic matter biodegradation and suspended solids removal, while the second is an unsaturated VSSF CW (VSSF USNAT CW) filled with fine gravel and an intermediate layer of coarse sand [2]. The VSSF UNSAT CW has a saturation zone, as the bottom 30 cm of the total 100 cm bed depth is ponded. The VSSF UNSAT CW is divided into three beds (A: 150 m^2^ planted with *Iris pseudacorus;* B: 150 m^2^ planted with *Scirpus lacustris*; and C&D: 300 m^2^ planted with *Typha latifolia* and *Juncus inflexus*) and two feeding lines (AB and C&D) to promote alternate and intermittent feeding, and thus, the proper reaeration of the beds [2]. The use of different CW configurations for the investigation of CEC removal has also been proposed in the recent literature [18].

### 2.3. Wastewater Influent Characteristics

Average influent wastewater quality regarding conventional macro-pollutants is shown in Table 1. Average pH of the influent wastewater was neutral to slightly alkaline throughout all the sampling campaigns, ranging from 7.4–7.8. Average total suspended solids (TSS) concentrations were high and ranged between 302–407 mg TSS/L, indicating that most of the organic matter was of a particulate nature. This was validated by a chemical oxygen demand (COD) analysis, where average total COD ranged between 621–748 mg COD/L, while soluble COD was only a small part of it (18–26%, on average). Influent nitrogen was mostly in the ammonium nitrogen (NH_4_-N) form, and it ranged between 51.1 and 65.3 mg NH_4_-N/L, on average, while no nitrates were detected, indicating that no significant agricultural run-offs were infiltrating the sewer system. Average total phosphorus (TP) and orthophosphates (PO_4_-P) concentrations were typical for domestic wastewater (6.6–11.1 mg TP/L and 5.6–7.4 mg PO_4_-P/L, respectively) [19]. Slight declinations were observed during stormwater events due to rainwater infiltration.

### 2.4. Operational Parameters of the Pilot System

The operational parameters of the system during the periods of the sampling campaigns are presented in Table 2. The most distinguishing characteristic of the three different periods was the influent wastewater flow. From April to December 2024, flowrate increased from 41.2 ± 3.6 m^3^/d to 59.2 ± 0.2 m^3^/d in May and, finally to 76.1 ± 6.6 m^3^/d in December. The conv-WWTP received, respectively, decreasing volume of wastewater that ranged from 63.4 ± 5.8 m^3^/d in April to 10.5 ± 3.2 m^3^/d in December. Apart from that, wastewater temperatures were similar in April and December, i.e., 16–17 °C, but it increased in May (equal to 19 °C). The varying flowrate in the pilot system affected the operational parameters of the subsystems. HRT of the UASB unit decreased during the three sampling periods from 24.0 ± 1.8 h in April to 12.8 ± 1.0 h in December. The increasing flowrate and the reported influent wastewater characteristics (Table 1), along with the performance of each subsystem, affected the operation loading of each subsequent stage in terms of organic loading rate (OLR), solids loading rate (SLR), and nitrogen loading rate (NLR), as described in Table 2. In general, flowrate increase induced an increased loading, but in December, loadings were only slightly increased––or even decreased––due to the good performance of the subsystems and the slight dilution of the influent (Table 1).

### 2.5. Sampling Campaigns

Three individual sampling events took place: the first in April 2024, the second in May 2024, and the third in December 2024. Seven sampling points were selected at each campaign: influent tank (pre-treated domestic wastewater, Table 1), effluent from the UASB, effluent from the saturated VSSF CW, effluent from the unsaturated VSSF CW (bed A), effluent from the unsaturated VSSF CW (bed B), effluent from the unsaturated VSSF CW (bed C&D), and effluent from the conv-WWTP. Three samples were collected over three consecutive days of each campaign, taking into account the HRT, and were analyzed for the detection of three benzotriazoles: xylytriazole (XTR), 5-chlorobenzotriazole (CBTR), 5-methyl-1H benzotriazole (5TTR); one benzothiazole: 2 hydroxybenzothiazole (OH-BTH); two endocrine disrupting chemicals: triclosan (TCS) and bisphenol A (BPA); and four non-steroidal anti-inflammatory drugs (NSAIDs): ibuprofen (IBU), naproxen (NPX), diclofenac (DCF), and ketoprofen (KFN) at each point during each campaign, resulting in a total of 72 samples (24 samples per campaign).

### 2.6. Organic Micropollutants Analytical Methods

Standards of XTR, CBTR, BPA, TCS, IBU, NPX, KFN, DCF (as sodium salt), deuterated BPA (BPA-d_16_), meclofenamic acid sodium salt (MCF), were supplied by Sigma–Aldrich (Saint Louis, MO, USA), 5TTR was obtained from Acros Organics (Geel, Belgium), and OH-BTH from Alfa Asar (Ward Hill, MA, USA). Methanol (MeOH), ethyl acetate (ETH), and acetonitrile (ACN) of high purity grade were purchased from Honeywell (Charlotte, NC, USA) and Fisher (Waltham, MA, USA), respectively. Strata-X cartridges for BTRs and OH-BTH (33u Polymeric Reversed Phase, 200 mg/6 mL) and Isolute C18 cartridges (500 mg/6 mL) for EDCs and NSAIDs were used for solid phase extraction (SPE) and were supplied by Phenomenex (Torrance, CA, USA) and Biotage (Uppsala, Sweden), respectively. Bis(trimethylsilyl)trifluoroacetamide (BSTFA) +1% trimethylchlorosilane (TMCS), pyridine and ultra-pure HCl (32% *w*/*w*) were also supplied by Sigma–Aldrich (Saint Louis, MO, USA).

Target EDCs (TCS, BPA) and NSAIDs (IBU, NPX, DCF, KFN) were analyzed by implementing the method developed by Samaras et al. [20]. Samples were filtered using 0.45 μm membranes supplied by Whatman (Dassel, Germany), acidified to the pH value of 2.5 ± 0.2 and then spiked with a 60 μL mix of an internal standard mixture (BPA-d16 for EDCs and MCF for NSAIDs) at a concentration of 600 ppb. This spiking resulted in a final amount of 36 ng of each internal standard per sample, used as surrogates. The samples were stored in the fridge (4 °C) covered by aluminum foil to protect them from the light until SPE took place. The eluates were evaporated to dryness using nitrogen gas (N_2_) and were derivatized using 10 μL pyridine and 50 μL BSTFA + 1% TMCS in a batch device (GALLENKAMP BKS-350) (Gallenkamp, Cambridge, UK) of 70 °C for 20 min. After the solutions were cooled to room temperature, a 1 μL injection was performed using a 7890A gas chromatograph (GC) coupled with a 5975C mass selective detector (MSD) by Agilent Technologies (Santa Clara, CA, USA). The chromatographic GC column was Agilent J&W HP-5ms Ultra Inert (Santa Clara, CA, USA). Finally, the quantification of analytes was performed using selected ion monitoring and the integration of the curves was carried out using ChemStation software version E.02.00.493 by Agilent Technologies (Santa Clara, CA, USA).

For the analysis of target benzotriazoles and OH-BTH, an aliquot of 100 mL was collected and filtered through membrane filters of 0.45 μm by Whatman (Dassel, Germany), acidified at pH 3.0 ± 0.1 with few drops of HCl, and stored at 4 °C. The analysis of the liquid samples included SPE and was based on the method developed by Asimakopoulos et al. [21]. For the chromatographic analysis, the mobile phase consisted of MilliQ grade water (acidified with 0.1% *v*/*v* formic acid) and ACN. Gradient elution was carried out as follows: 25% ACN to 75% ACN in 15 min, hold for 9 min, and then decrease to 25% ACN in 1 min. Before each run, equilibration was performed for 10 min with 25% ACN. Εach run had a total duration of 35 min and a flow rate of 0.5 mL/min [22]. The model of High-Performance Liquid Chromatography (HPLC) was Shimatzu (Kyoto, Japan) LC20-AD with an SPD-M20A diode array detector (DAD) (Wetzlar, Germany) using signal at 254 nm and a SIL-20AC auto sampler (Shimadzu Corporation, Kyoto, Japan). The model of column was Zorbax SB-C18 4.6 mm 150 mm (5 mL connected with a pre-column Zorbax SB-C18) by Agilent (Santa Clara, CA, USA) and they were heated at 35 °C with a CTO-20AC column oven by Shimatzu (Kyoto, Japan). The identification of the target compounds in the samples was accomplished by retention times and by comparing their UV spectrum in the standard solutions and in the samples. Finally, the integration of the curves was carried out though LC solution software version 1.21 by Shimadzu (Kyoto, Japan). Satisfactory recoveries and precision of the analytical procedures were achieved. Information for the analytical method can be found in the publication of Mazioti et al. [22].

### 2.7. Data Analysis

Removal efficiency between different stages of the pilot system was calculated using Equation (1), while negative removals were set to zero:

(1) $$Re\left(\%\right)=\frac{C_{in}-C_{out}}{C_{in}}*100$$

where $C_{out}$ is the concentration at the outlet of the considered treatment stage and $C_{in}$ is the influent concentration, respectively.

Total removal efficiency of each one of the pilot’s configurations and conventional WWTP system was calculated through Equation (1), where: $C_{out}$ is the final effluent concentration and $C_{in}$ is the influent concentration.

Contribution of each internal stage of the pilot in total removal efficiency was calculated using the following Equation:

(2) $$Re\left(\%\right)=\frac{C_{in,i}-C_{out,i}}{C_{in}}*100$$

where i corresponds to each individual system (UASB, VSSF SAT CW, VSSF UNSAT A, VSSF UNSAT B, VSSF UNSAT CD) and $C_{in}$ is the inlet concentration of the whole system.

One-way ANOVA and Tukey’s Honestly Significant Difference tests were applied to the temperature, CECs’ influent concentration, mass and removal efficiency data to identify important differences among the results, using a significance level of 95%. Statistical analyses were conducted using SPSS version 30 by IBM (Armonk, NY, USA). The occurrence of statistically significant differences among the three periods (output) was examined for the following inputs: (a) temperature, (b) influent concentrations of each target CEC, (c) influent masses of each target CEC, (d) the overall removal of each CEC by the pilot-UNSAT A line and (e) the conv-WWTP line separately, and (f) the contribution of each subsystem of the pilot line (i.e., UASB, VSSF SAT CW, and VSSF UNSAT CW line A) to the overall pilot line efficiency of each CEC. Moreover, the significant differences in performance among the pilot-UNSAT A line and the conv-WWTP (output) were evaluated for each period and each compound separately (input). Comparison among systems expanded on the assessment of any significant differences in the overall CEC removal efficiency among pilot line’s three alternatives, i.e., pilot-UNSAT A, pilot-UNSAT B, and pilot-UNSAT CD (output), for each period and compound, separately (input).

### 2.8. Risk Assessment Analysis

Risk assessment analysis was performed by calculating the risk quotient (RQ) of each organic micropollutant for three different aquatic organisms: fish, *Daphnia magna*, and algae, based on a study by Thomaidi et al. [23]. RQ values were calculated by dividing the maximum environmental concentration (MEC) by the Predicted No Effect Concentration (PNEC), as shown in Equation (3):

(3) $$RQ=\frac{MEC}{PNEC}$$

MEC was associated with two scenarios. The first scenario was the base scenario, where MEC was considered equal to the average measured concentration in the final effluent of each tested system (conv-WWTP, pilot-UNSAT A), which is more realistic. In the second scenario, which is the worst case, MEC was set equal to the maximum measured concentration in the final effluent in each system. A dilution factor was not considered, given that the nearby water body is an ephemeral river and effluent is only diluted during the rainy season. PNEC value was obtained by dividing the lowest $LC_{50}$ or $EC_{50}$ value with an assessment factor of 1000 (Equation (4)), since short-term toxicity data were used. $LC_{50}$ or $EC_{50}$ values were found in the literature, where researchers either performed lab toxicity tests, or they used the ECOSAR prediction model [23]. More information about the values of $EC_{50}$ or $LC_{50}$ that have been used can be found in Supplementary Material (Table S4).

(4) $$PNEC=\frac{LC_{50}\;or\;EC_{50}}{1000}$$

An ecotoxicological risk is indicated when the RQ value is greater than 1, while no risk is anticipated when the RQ value is less than 1. $RQ_{mix}$ or cumulative RQ was calculated by adding RQ individual values of all target analytes.

## 3. Results and Discussion

### 3.1. Conventional Pollutants Removal Efficiency of the Pilot System

**Figure 2:**
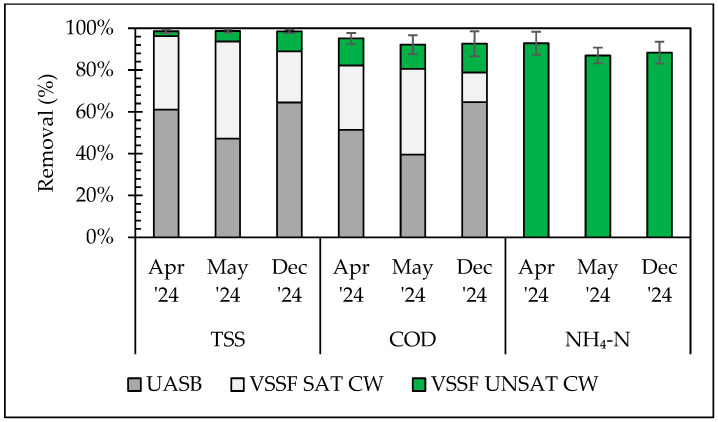
Pilot system efficiency (average ± SD) for the abatement of TSS, COD, and NH_4_-N (contribution of each subsystem to the overall removal). Error bars represent standard deviation of the total removal efficiency of the pilot system.

The pilot system was capable of almost complete elimination of TSS (99% ± 1%) at all periods (Figure 2). This was attributed to the simultaneous mechanisms of sedimentation, entrapment in the biomass and hydrolysis in the UASB and the VSSF SAT CW, while the unsaturated VSSF CW enhanced the elimination through filtration in the intermediate coarse sand layer [24]. The UASB reactor’s TSS removal was equal to 61% ± 16% and 64% ± 12% in the 1st and 3rd periods, respectively, only slightly decreasing and becoming less stable in the 2nd period (47% ± 23%). Considering that upflow velocity was kept stable by regulating internal recirculation, the performance drop was attributed, besides HRT decrease, to the increase in temperature and TSS concentration in the influent. It has been reported that the latter can cause turbulence inside the reactor due to increased methane production [25]. The VSSF SAT CW was able to cope with the decreased UASB TSS removal in the 2nd period, increasing its contribution to the overall removal. Similarly, the VSSF UNSAT CW performed well under increased SLR in the 3rd period.

Overall COD removal efficiency was very high (up to 95% ± 3% in the 1st period), only slightly decreasing to 92% ± 6% in the 3rd period under the maximum flowrate. The UASB removed COD from the raw domestic wastewater via hydrolysis and anaerobic degradation [26], with efficiency linked to TSS removal. A decrease in performance during the 2nd period (40% ± 16%) was followed by recovery in the 3rd (65% ± 8%). The VSSF SAT CW contributed most to COD removal in the 2nd period (41% ± 12%), but dropped to 14% ± 8% in the 3rd, likely due to reduced HRT (Table 2). Considering the high TSS removal efficiency of the first two subsystems (Figure 2), the OLR applied in the VSSF UNSAT CW was attributed to soluble organic matter. The VSSF UNSAT CW maintained stable contribution to COD removal (12% ± 2%–14% ± 4%) across periods, thanks to appropriate (according to existing guidelines [19]) organic loading and resting periods.

Ammonium nitrogen, as anticipated, was not removed by the UASB and the VSSF SAT CW. On the other hand, minor release of ammonium nitrogen was observed due to mineralization [27]. Ammonium nitrogen was successfully removed in the VSSF UNSAT CW at a range of 87% ± 4% (2nd period)–93% ± 6% (1st period), mostly through aerobic nitrification outperforming comparable systems [27] that achieved 61% removal with partially saturated VSSF CWs under a similar loading (6 g N/m^2^-d).

The conv-WWTP was monitored by the water utility and its effluent met the requirements from the Directive 91/271/EEC regarding small settlement WWTPs (<10,000 inhabitants) discharging in non-sensitive water bodies, i.e., BOD_5_ < 25 mg/L, COD < 125 mg/L and TSS < 60 mg/L (https://eur-lex.europa.eu/eli/dir/1991/271/oj/eng, accessed on 9 April 2025).

### 3.2. Occurrence of Target CECs in Influent Wastewater

The target CECs were detected in all nine samples of pilot system influent (frequency of detection = 100%), except OH-BTH (frequency of detection = 67%). Average value, standard deviation, and range (min–max) are presented in Table 3 for all samples (*n* = 9). The corresponding values for each individual sampling event are available in Supplementary Material (Table S1). Influent concentrations of IBU, TCS, BPA, DCF, KFN, and 5TTR did not differ significantly across the different periods (*p* > 0.05). In contrast, NPX concentrations were significantly lower during the 3rd period compared to the 1st and 2nd periods (*p* < 0.05). Conversely, influent concentrations of both CBTR and XTR were significantly higher during the 3rd period relative to the 1st and 2nd (*p* < 0.05). OH-BTH displayed a distinct pattern, with the highest influent concentrations observed during the 2nd period, followed by the 1st, while it was not detected at all during the 3rd period (*p* < 0.05). The highest average concentration of all periods was observed for CBTR (8608 ± 7504 ng/L) and the lowest for TCS (444 ± 166 ng/L). The high NSAIDs detection frequencies are in line with those reported by Česen et al. in Slovene [28] and can be attributed to their widespread consumption for relieving pain and fever. In contrast to our results, Česen et al. [28] found lower detection frequencies for BPA (73.1%) and TCS (50%) [28]. BPA, TCS, IBU, NPX, DCF, and KFN concentrations were higher than those reported by Česen et al. [28]. Very low concentrations of TCS have also been reported in China; for example, Wang et al. [29] were unable to detect TCS in most of their samples, with the median concentration falling below the detection limit. In contrast, significantly higher concentrations were observed in the U.S.A., ranging from 179 to 2523 ng/L, according to a study by D’Alessio et al. [30]. XTR and CBTR concentrations were significantly higher than those observed in Greek municipal wastewater [31,32], whereas the levels of 5TTR and OH-BTH were comparable to those reported by Koukoura et al. [32]. Representative influent concentrations of the target CECs in municipal WWTPs are summarized in Table 4 below.

Among different geographical regions, NSAID concentrations fluctuate in influents due to their usage patterns [33]. NPX and DCF had similar average influent concentrations, approximately 5000 ng/L, while KFN exhibited the lowest influent concentration within the NSAIDs group, ranging from 282 ng/L to 2067 ng/L. Consistent with our study, NPX was found at an average concentration of nearly 5000 ng/L in WWTP influents in Sweden, but DCF was significantly lower (230 ng/L) compared to our findings [34]. KFN was detected in China at concentrations ranging from 13 ng/L to 1030 ng/L [29] and 100.6 to 7881 ng/L [29,35], showing a similar trend to our case. Although IBU had the lowest concentration among the target NSAIDs in the study by Styszko et al. [36], at 8100 ± 800 ng/L, it was the highest among the NSAIDs in our study, with a concentration of 6595 ± 1841 ng/L, which is comparable to their findings. However, IBU was detected at nearly 100,000 ng/L in WWTP influent in the U.S.A. [30], while another study conducted in Greece reported concentrations ranging from 2800 to 25,400 ng/L [37].

Average daily mass loads were calculated for NSAIDs (as mg per 1000 inhabitants) in influent wastewater and they are presented in Table 3. The highest average mass load was observed for IBU (1055 mg/d/1000 inh) and the lowest for KFN (119 mg/d/1000 inh). The presence of NSAIDs in wastewater is primarily due to human excretion, meaning their mass loads can serve as indicators of consumption patterns. Mass loads of IBU and NPX (843 mg/d/1000 inh) were much lower compared to Sweden (3600 and 2560 mg/d/1000 inh for IBU and NPX, respectively) as reported by Zorita et al. [34]. However, mass load for DCF was higher (874 mg/d/1000 inh) compared to Sweden (122 mg/d/1000 inh). Papageorgiou et al. [38] reported similar values for NPX (714 mg/d/1000 inh) but higher values for DCF (2318 mg/d/1000 inh) and KFN (386 mg/d/1000 inh) in Volos, Greece.

**Table 4:** Influent concentrations of the target CECs in municipal wastewater treatment plants.

Compound	Ιnfluent Concentration (ng/L) in Municipal WWTPs	Ref.
IBU	Greece: 2800–25,400, 12,500 (range, mean)	[37]
	Sweden: 6900 ± 900 (mean ± SD)	[34]
	USA: 16,433–96,519 (range)	[30]
	China: 268–2240, 628, 811 (range, median, average)	[29]
	Slovenia: 4.61–77.9, 43.9, 100% (range, average, frequency of detection)	[28]
NPX	Greece: n.d.–2000, 1500 (range, mean)	[37]
	Sweden: 4900 ± 480 (mean ± SD)	[34]
	USA: 15,544–45,386 (range)	[30]
	China: 1.63–20.4, 11.0, 11.4 (range, median, average)	[29]
	Slovenia: 81.3–1290, 361, 100% (range, average, frequency of detection)	[28]
DCF	Greece: n.d.–3900, 2000 (range, mean)	[37]
	Sweden: 230 ± 9 (mean ± SD)	[34]
	China: 128.6–1027.1 (range)	[35]
	Slovenia: 2.09–48.5, 15.5, 96.2% (range, average, frequency of detection)	[28]
KFN	China: 100.6–7881.0 (range)	[35]
	China: 13.0–1030, 236, 299 (range, median, average)	[29]
	Slovenia: 0.534–692, 55.3, 84.6% (range, average, frequency of detection)	[28]
TCS	Greece: n.d.–1000, 800 (range, mean)	[37]
	USA: 179–2523 (range)	[30]
	China: BDL ^1^–62.9, BDL, 5.30 (range, median, average)	[29]
	Slovenia: 5.27–9.68, 6.64, 50% (range, average, frequency of detection)	[28]
BPA	Slovenia: 11.2–489, 95.7, 73.1% (range, average, frequency of detection)	[28]
	China: 836.9 ± 87.2 (mean ± SD)	[39]
5TTR	Australia: 6758 ± 1438 (mean ± SD)	[40]
	Greece: 3579 ± 179 (mean ± SD)	[32]
CBTR	Australia: 1196 ± 301 (mean ± SD)	[40]
	Greece: 3875 ± 833 (mean ± SD)	[32]
XTR	Greece: n.d.–55.3, 27, 79% (range, average, frequency of detection)	[31]
	Greece: 2767 ± 1106 (mean ± SD)	[32]
OH-BTH	Greece: 256–908, 503, 100% (range, average, frequency of detection)	[31]
	Greece: 7076 ± 2275 (mean ± SD)	[32]

^1^ Below detection limit.

### 3.3. Influence of Operational Conditions on Total Removal of Pilot with VSSF UNSAT CW (Line A) and the Conventional WWTP

This section presents total removal efficiencies of the target CECs for each sampling campaign, highlighting the influence of operational parameters on their removal in both the novel pilot system (UASB-SAT-UNSAT A) and the conventional WWTP (conv-WWTP). The comparison focuses on line A (UNSAT A) only of the unsaturated CWs, as differences among the parallel UNSAT lines were minimal, which is further discussed in Section 3.5. Based on the results and ANOVA analysis (*p* < 0.05), the pilot system exhibited a significant difference between the 1st and 3rd period for NPX removal and during the 1st and 2nd period for XTR removal. It resulted in 76% ± 5% NPX total removal efficiency when the flowrate was 40 m^3^/d during the 1st sampling period. This increased to 88% ± 3% when the flowrate doubled to 80 m^3^/d during the 3rd period. Even though the hydraulic load was higher in the 3rd period, the influent mass load of NPX remained similar (284 ± 74 mg/d, Table S2) to that of the 1st period (248 ± 30 mg/d, Table S2). Increased influent loads can lead to saturation of the medium, restrained microbial and plant metabolic activities, and antimicrobial effects of working microbiota, all of which can reduce removal efficacy, as reported by Chand et al. [41]. Based on these observed effects of influent strength, the lower concentration of NPX during the 3rd period compared to the 1st may have positively influenced microbial degradation and sorption [41,42], especially since temperature and NPX mass remained statistically similar (*p* > 0.05). Although NPX concentration and mass during the 2nd period were similar to those in the 1st, the removal efficiency was not significantly different from that of the 3rd period (*p* > 0.05), possibly due to the positive impact of the higher temperatures [42] (*p* < 0.05) on the removal processes during the 2nd period compared to the other two periods. XTR removal efficiency was greater than 91% during the 1st period (40 m^3^/d) and decreased to 81% ± 4% when the flowrate was 60 m^3^/d (2nd period). XTR concentration during the 1st and 2nd periods was very similar (2.7 ± 0.2 and 2.7 ± 0.5 ng/L, respectively), but the mass of the substance was higher in the 2nd period. However, this did not appear to affect the overall removal efficiency of the substance, as in the 3rd period both the mass and concentration values (11.8 ± 1.9 ng/L) were significantly higher, yet this did not statistically impact XTR removal efficiency (89% ± 4%). The behavior of XTR needs further investigation.

The conventional WWTP showcased significant differences (*p* < 0.05) in IBU, NPX, and DCF removals between the 3rd period and the 1st and 2nd periods; for 5TTR, significant differences were observed between the 1st period and the 2nd and 3rd; and for BPA, between the 2nd and 3rd periods. These results indicate a notable improvement in IBU, NPX, and DCF removal (97% ± 2%, 86% ± 2% and 88% ± 4%, respectively) during the 3rd period, whereas 5TTR was completely removed both at the 2nd and 3rd period. BPA removal was also ameliorated during the 3rd period (63% ± 9%). This superior performance of the 3rd period for the conventional activated sludge system could be attributed to the increase of HRT and SRT [43] induced by the very low flowrate (Table 2), promoting aerobic biodegradation through oxidation. CEC removal at WWTPs varies significantly in the literature. However, according to a simple classification by Luo et al. [44], IBU, NPX, TCS, and BPA can be considered highly removed (>70%), while KFN is classified as moderately removed (40–70%), and DCF as poorly removed (<40%).

Comparing the novel pilot system with the conventional WWTP for each compound and each sampling period, the pilot system demonstrated statistically important higher removal efficiency for eight CECs at least for one sampling period (see Table S3 in Supplementary Material). These results underscore the enhanced performance of the integrated UASB—CWs system in eliminating micropollutants, highlighting the effectiveness of this synergistic approach. The main reasons behind this could be: (a) the higher HRT [43] that is applied to the pilot system UASB-CWs compared to the conv-WWTP and (b) the variety of microenvironments and physicochemical conditions that co-exist in CWs, and especially when different configurations are being combined [14]. This heterogeneity supports a variety of metabolic and co-metabolic pathways [41,42], enhancing the degradation of pharmaceuticals and personal care products in contrast to the conv-WWTP where typically more uniform physicochemical conditions are maintained, limiting the range of degradation mechanisms available [45].

### 3.4. Contribution of Each Treatment Stage of the Pilot System to the Overall Performance and Removal Mechanisms

This section includes the results of the removal efficiency comparison for each period at each stage of the pilot system, along with a discussion of the potential removal pathways. The removal of each stage was calculated as a part of the total removal (contribution to the overall removal efficiency), according to Equation (2) (Section 2.7). ANOVA analysis showcased that there are no significant differences (*p* > 0.05) among the three sampling periods for UASB, VSSF SAT CW, and VSSF UNSAT CW (line A) for all EDCs and NSAIDs, revealing that the changes in HRT, OLR, SLR, and NLR in these units did not influence EDCs and NSAIDs removal. However, removal through the UASB unit was significantly (*p* < 0.05) higher during the 3rd period compared to the other two periods (1st and 2nd), respectively, for the target benzotriazoles: 5TTR, CBTR, and XTR, while OH-BTH removal was outstandingly higher (*p* < 0.05) during the 1st period than the 2nd. As far as VSSF SAT CW is concerned, significant differences in removal efficiency occurred between the 2nd period and the other two periods (1st and 3rd) for 5TTR, with enhanced removal during the 2nd period. Similarly, XTR removal showed significant differences between all periods, increasing in the order of 3rd < 2nd < 1st. OH-BTH removal differed significantly between the 1st and the 2nd periods. Statistically, there was no important difference in the removal efficiency of CBTR across the three periods at the SAT unit. It should be noted that OH-BTH was not detected at all in the 3rd period, neither in the influent, nor at any effluent. In the VSSF UNSAT CW, a significant decrease in the removal efficiency of 5TTR was observed during the 3rd period compared to the 1st and 2nd periods. Similar to the VSSF SAT CW, OH-BTH removal efficiency in the VSSF UNSAT CW differed significantly between the 1st and 2nd periods.

**Figure 3:**
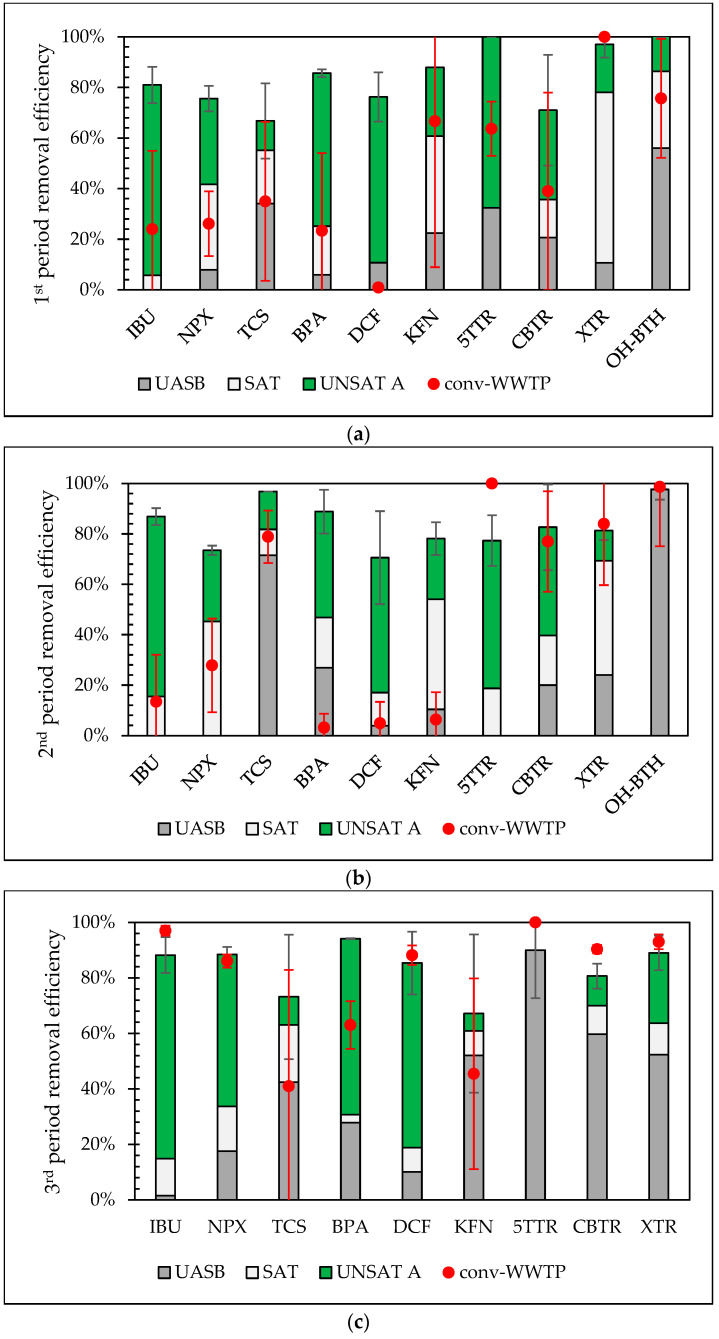

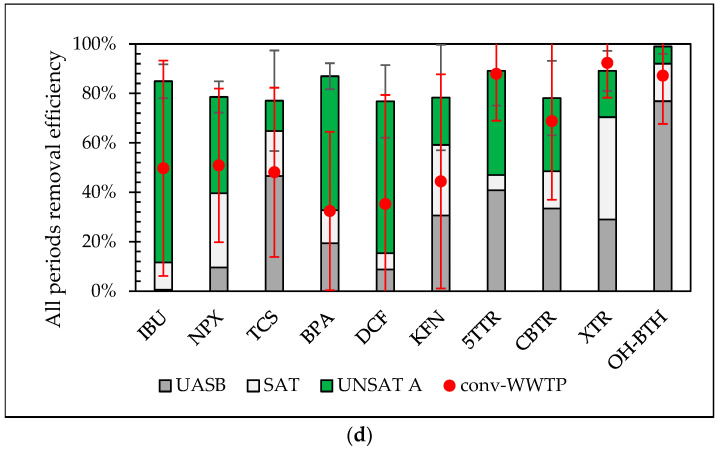
Contribution (average values) of UASB, VSSF SAT CW, and VSSF UNSAT CW (line A) to the target CECs’ removal efficiency of the pilot system in comparison with the removal efficiency of the conventional WWTP for the 1st (**a**), 2nd (**b**), and 3rd (**c**) periods, as well as for the whole monitoring period (**d**). Error bars represent standard deviation of the total removal efficiency of each system (pilot, conv-WWTP).

According to the results, 5TTR, CBTR, XTR, TCS, and KFN were only partially removed, by UASB system (Figure 3). Concerning removal of specific target compounds, their average removals ranged between 29% (XTR) and 47% (TCS). BPA was removed at a lower rate of 19%, while NPX and DCF were removed at minimal rates of approximately 10%. IBU showed no removal, with some samples even displaying negative values (that were set to zero). On the contrary, benzothiazole was removed at higher rates in the UASB system, with an average removal efficiency of 77 ± 25% for OH-BTH (Figure 3).

Previous studies have shown that benzotriazoles and benzothiazoles were biodegraded under anaerobic conditions [22,40,46]. Liu et al., 2011 [46] observed 61% and 71% reduction for 5TTR and CBTR, respectively, in a laboratory-scale reactor under strictly anaerobic conditions, attributed to biodegradation. Among the removal mechanisms occurring in the UASB reactor, the attenuation of target compounds is likely attributed to anaerobic biodegradation, as the sorption process appears to be insufficient [46,47]. Anaerobic sludge holds a limited amount of organic matter, which reduces its capacity to sorb the target micropollutants. Furthermore, benzotriazoles and benzothiazole are polar, generally hydrophilic molecules that exhibit poor affinity for the particulate phase, as indicated by their low LogKow coefficient values (1.44–2.13) [48,49].

TCS is a lipophilic compound, (LogKow coefficient = 4.80 [50]) and it has the tendency to bind to suspended organic matter. In addition, its presence in wastewater that has a pH value of 7–8 is in its protonated form (pKa = 7.90 [50]). Thus, its sorption into UASB biosolids, along with biodegradation [51], can contribute to its removal, explaining the important contribution of this stage on its overall removal. On the other hand, the anionic form of NSAIDs (pKa = 4–5 < pH = 7–8 [52]) is responsible for their repulsion from sludge blanket, which is also negatively charged, leading to their remaining in the liquid phase inside the UASB reactor [53,54], while anaerobic biodegradation can take place at a low or high level depending on the compound. Anaerobic treatment in the UASB unit demonstrated low removal rates for IBU (19%), NPX (17%), DCF (10%), and BPA (1.5%) as reported by Vassalle et al. [54], which is in alignment with the results of this work. In that study, the UASB unit served as the initial stage of a combined treatment system, followed by high-rate algal ponds. Queiroz et al. [53] also observed insufficient removal of BPA and DCF. Surprisingly, Martin et al. [55] found 100% average removal of KFN by UASB treatment. Biotransformation was the primary removal mechanism for IBU, NPX, and DCF in UASB unit, as reported by Alvarino et al. [56]. In this work, NPX recorded very high removal (more than 90%), in contrast to our results; however, the low-rate efficiency of the UASB unit towards IBU and DCF (≤20%) aligns with our findings. The recalcitrant nature of DCF and IBU in anaerobic environments is supported by their low biodegradation rates [53].

Low to medium average removal efficiencies were observed in the VSSF SAT CW, with some samples displaying negative values (5TTR). Specifically, at this stage, OH-BTH removal efficiencies were up to 30%, while, for 5TTR and CBTR, did not exceeding 20%. XTR removal efficiency was satisfactory during the 1st and 2nd periods, with removal efficiencies of 67% ± 4% and 45% ± 4%, respectively. However, during the 3rd period, the increased flow rate led to a significant decline in removal efficiency, decreasing to 11% ± 9%. The contribution of saturated VSSF CW to the overall removal efficiency across all periods did not exceed 15% for 5TTR, CBTR, and OH-BTH. Regarding the unsaturated VSSF CW, BTR and BTH removal was more effective compared to saturated, except for XTR (Figure 3). During the 1st period, the contribution of the unsaturated VSSF CW to the overall removal efficiency of these compounds ranged from 14% (OH-BTH) to 68% (5TTR). In the 3rd period, its contribution was 11% (CBTR) to 25% (XTR), excluding OH-BTH—which was not detected—and 5TTR, which had already been completely removed in the previous treatment stages. 5TTR, XTR, and OH-BTH effluent concentrations were nearly zero, indicating this stage’s substantial contribution to the overall removal efficiency of the system. As shown in Figure 3, CBTR exhibited low removal efficiencies across all treatment units. The increased flow rate during the 2nd period did not appear to significantly affect removal performance of the compounds compared to the 1st period. Nevertheless, OH-BTH was completely removed during the first treatment stage (UASB reactor), preventing the evaluation of the removal performance of the unsaturated VSSF CW during this period.

The low average contribution of saturated VSSF wetland removal across all periods in total removal of IBU, DCF, and BPA (<15%) differs significantly from the average contribution of the unsaturated VSSF wetland (>50%). In terms of NPX, the difference between the contribution of saturated and unsaturated CW in its removal was marginal, since it was 30% and 39%, respectively. Τhe contribution of the saturated VSSF CW to TCS and KFN removal was slightly higher (18% for TCS and 29% for KFN, respectively), than that observed for the unsaturated wetland (12% for TCS and 19% for KFN, respectively). It should be kept in mind that these values are not the individual rates, but the accumulated rates.

Saturated CWs often underperform in micropollutant removal due to limited oxygen availability, lower adsorption capacity, and insignificant photodegradation due to subsurface conditions [14,57]. Several studies have explored the effectiveness of CWs in removing BTRs and BTHs. Felis et al. [58] operated an unsaturated SSF CW combined with artificial sunlight to assess BTR and BTH removal efficiency. Their findings revealed that integrating sunlight-induced processes with SSF treatment greatly improved removal efficiency of the target compounds, with BTR exhibiting particularly high elimination rates (99.7%). In another study, Matamoros et al. [59] examined the effect of seasonal variation in OH-BTH and 5TTR removal efficiency in a full-scale FWS CW. The results showed that 80% of OH-BTH was removed during the warmer months, while the removal rate for 5TTR was 50%.

For IBU, DCF, and BPA, high redox conditions and aerobic microbial processes play an important role in their depletion [56,60,61,62]. Matamoros et al. [63] also found that IBU, NPX, and DCF were removed at higher rates (individual rates) in unsaturated VSSF wetland (99% ± 1%, 89% ± 5%, 73% ± 3%) than in saturated VSSF wetland (55% ± 1%, 62% ± 3%, 53% ± 2%). The marginal difference between the contribution of VSSF SAT CW and VSSF UNSAT CW can be attributed to the effective degradation of NPX under both anaerobic and aerobic conditions [56,62,64]. The recalcitrant nature of KFN, attributed to its structural characteristics, including two extended aromatic rings [65], is evident in its fluctuations across the treatment stages of the current study. However, the high average total removal (79%) is likely driven by biodegradation [66] that can take place through anaerobic and aerobic microbes present at different stages. The UASB reactor and the saturated VSSF CW contribute equally to the overall process (31% and 29%, respectively), while lower contribution is observed for the unsaturated wetland (19%). Thus, the main mechanisms are assumed to be primarily anaerobic biotransformation [51] in the UASB unit and saturated wetland and aerobic biodegradation [64] in the unsaturated wetland. The saturated and unsaturated wetlands showed similar patterns in their contributions to TCS removal. As already explained, sorption into organic matter can take place due to the hydrophobic nature of this compound, while its anaerobic and aerobic breakdown by microbes may occur as well [51,60,64]. In addition, biodegradation of TCS and DCF under anoxic conditions can occur through dehalogenation [67], in particular in the saturated zone at the bottom of the VSSF UNSAT CW, where nitrites are present at high concentrations due to ammonium oxidation in the upper zones.

Taking into account the average contribution of each treatment stage across all periods, UASB played an important role in TCS and OH-BTH removal. The SAT stage participated remarkably in XTR removal, while the UNSAT stage was an essential contributor to IBU, BPA, and DCF removal. The UASB and UNSAT units contributed almost equally to the removal of 5TTR and CBTR, while SAT and UNSAT showed similar contributions to the removal of NPX. In the case of KFN, both the UASB and SAT units were main contributors to its removal.

### 3.5. Comparison of the VSSF UNSAT CW Lines (A, B, C&D) Performance

**Figure 4:**
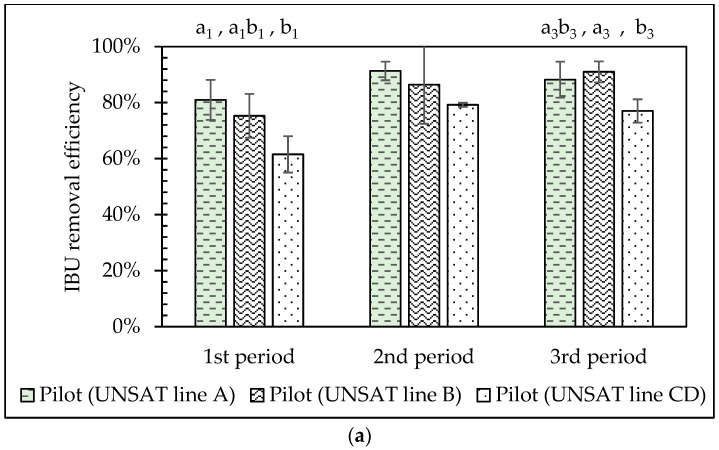

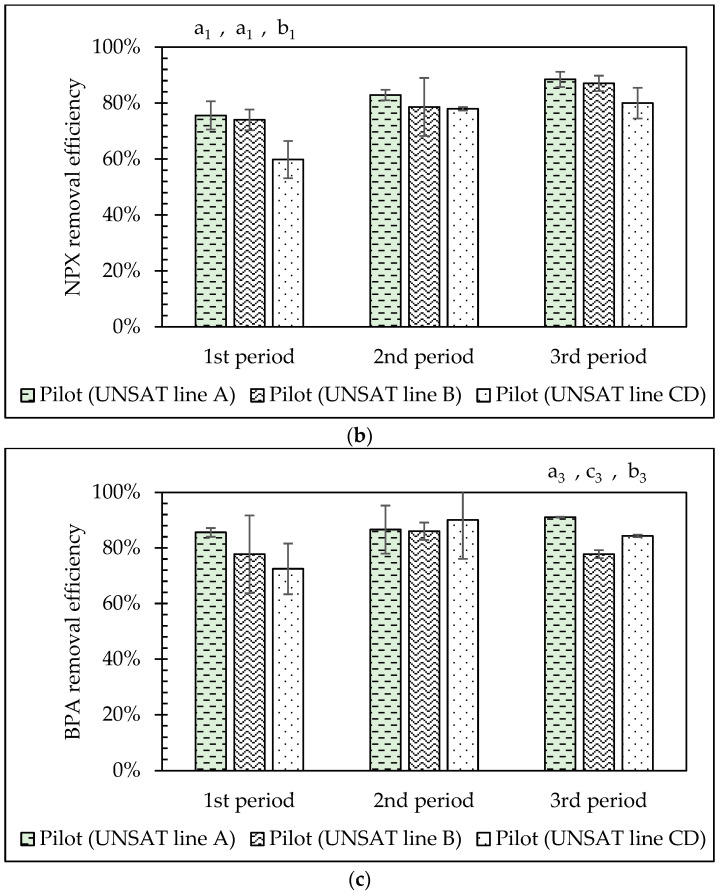
Removal efficiency (average ± SD) of IBU (**a**), NPX (**b**), and BPA (**c**) for which significant (*p* < 0.05) performance differences were observed in the pilot system. Small indexed letters represent the different significance classes, where applicable.

ANOVA analysis between the three different UNSAT lines (A, B, C&D) showed statistically significant differences (*p* < 0.05) only for IBU, NPX, and BPA, and only during specific period(s), as shown in Figure 4. Regarding the removal of IBU, UNSAT A was more efficient than UNSAT C&D during the 1st period, while UNSAT B outperformed UNSAT C&D during the 3rd. For NPX, UNSAT A and UNSAT B showed better removal than UNSAT C&D during the 1st period. BPA removal was highest in UNSAT A during the 3rd period (91%), followed by C&D (84%) and B (78%). The use of different plant species in each VSSF UNSAT CW line appeared to have limited effect on CECs’ removal efficiency.

Apart from phytoremediation, plants contribute to oxygen supply into the support matrix though their respiration by macrophyte roots and rhizome systems, which can also provide substratum for the growth of aerobes capable of degrading the micropollutants [41]. *Typha latifolia, Juncus inflexus, Iris pseudacorus*, and *Scirpus lacustris* have fibrous root systems and rhizomes that spread horizontally. However, Juncus has a shallower root system and a less dense rhizome compared to the others, which may have influenced oxygen input and aerobic microbial activity. Predictions of plant uptake and translocation to the shoots that are based only on LogKow should be approached with caution, especially for ionizable compounds. This is because their plant uptake is influenced both by the chemical pKa and the pH of the solution, and the permeability ratio between neutral and ionic forms of the molecules [52]. According to previous studies, the presence of plants (*Colocasia* sp.) in mesocosm-scale VSSF CWs improved IBU removal at different inlet loads [41]. Plant uptake and biodegradation were the removal mechanisms for IBU, whereas photodegradation was the predominant removal mechanism for DCF, TCS, and NPX through hydroponic planted reactors with *Salvinia molesta*, *Lemna minor*, *Ceratophyllum demersum*, and *Elodea canadensis* [68]. However, photodegradation is unlikely to occur in the pilot system of this study, since both the SAT and UNSAT wetlands are VSSF systems. Plants did not enhance the removal efficiency of BPA in the research of Papaevangelou et al. [60], where *Phragmites australis*, *Typha latifolia*, and unplanted VSSF wetlands were examined, recording removal efficiencies of 44.7%, 56.6%, and 57.2%, respectively. On the other hand, DCF was taken up efficiently by *Phragmites australis* (15 ng/g FW), while KFN and TCS (125 ng/g FW and 90 ng/g FW) were translocated to the aerial part of *Salix matsudana* [69].

### 3.6. Risk Assessment

**Figure 5:**
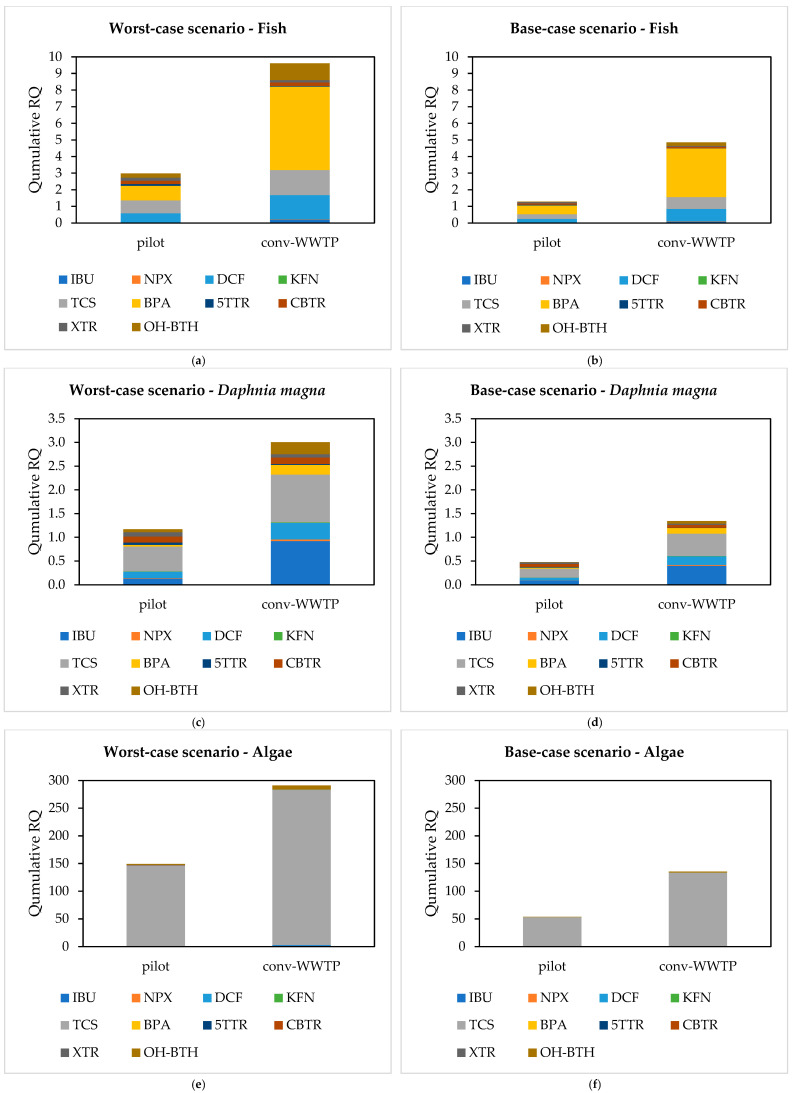
Cumulative risk quotient (RQ) of the studied CECs for worst-case and base-case scenarios for fish (**a**,**b**), *Daphnia magna* (**b**,**c**), and algae (**e**,**f**), respectively.

This section presents the results of the environmental risk assessment for three representative aquatic organisms—fish, *Daphnia magna*, and algae—under both base-case and worst-case exposure scenarios. As illustrated in Figure 5, the pilot treatment system demonstrated a lower ecological risk profile compared to the conv-WWTP. For IBU, NPX, DCF, KFN, BPA, 5TTR, CBTR, and XTR, RQ values remained below 1 for all three trophic levels under both exposure scenarios when the pilot treatment system was applied (Table S5). In the case of conv-WWTP, RQ values exhibited no risk for NPX, KFN, 5TTR, CBTR, and XTR towards the two scenarios and the studied aquatic organisms (Table S6). Among the compounds studied, BPA exhibited the highest individual RQ values for fish in both treatment systems and scenarios (5.02 and 2.93 for the conv-WWTP, 0.87 and 0.53 for the pilot system, under worst-case and base-case scenarios, respectively). For *D. magna* and algae, TCS consistently presented the highest RQs for both scenarios and treatment systems.

Focusing on the pilot system, TCS exhibited RQ values lower than 1 for fish and *D. magna*, but exceeded the threshold of 1 for algae in both scenarios, indicating a potential risk specifically to primary producers. Similarly, OH-BTH posed a significant risk only to algae and exclusively under the worst-case scenario.

Despite generally lower individual RQs, the cumulative risk (summed RQ values across all assessed compounds) exceeded 1 in the worst-case scenario for all organisms and both treatment systems, indicating potential combined effects. Even if the toxicity of a single substance is low and acute effects may be unlikely, important ecotoxicological effects could occur [70] as a CECs mixture. Under the base-case scenario, cumulative RQs for the pilot system were calculated as 1.29 for fish, 0.48 for *D. magna*, and 53.70 for algae. The elevated cumulative RQ for algae was predominantly driven by TCS, which showed individual RQ values of 145.86 and 52.81 under worst-case and base-case scenarios, respectively, underscoring its potential ecological toxicity. These findings align with previous studies [28,36], which also identified TCS as a high-risk contaminant in surface waters despite its relatively low environmental concentrations.

## 4. Conclusions

Of the ten target CECs, all were detected in the influent of the pilot system except for the OH-BTH during the 3rd period. The integrated UASB—two-stage CW system achieved high performance regarding the abatement of the target CECs across all periods. The removal efficiencies achieved ranged from 78% ± 21% (KFN) to practically 100% (OH-BTH) for a total of nine samples (six in the case of OH-BTH). The performance of the pilot system was found to be robust, since no major statistical differences were observed for the majority of the target CECs among the three different operational conditions that were tested. The only exceptions were for the NPX and XTR. The integrated pilot system was found to provide enhanced treatment in comparison to the conventional WWTP for all the target CECs, except for 5TTR and XTR, for which the treatment level was comparable. The different plants that were used in the three lines of unsaturated VSSF CW did not appear to affect the performance, as no significant differences were observed. Though the investigation did not include a side-by-side comparison of the treatment systems, most of the target CECs were successfully treated by UASB, saturated and unsaturated CWs, while IBU, BPA, and DCF were mostly removed in the unsaturated CW. The risk assessment revealed a lower ecological risk profile from the treated wastewater discharge of the pilot system compared to the conventional WWTP. However, cumulative Risk Quotient under the worst-case scenario indicated potential risks across all trophic levels, with TCS emerging as the predominant contributor for *Daphnia magna* and algae, particularly posing a significant threat to primary producers (algae). Future research should focus on the elucidation of the underlying removal mechanisms and identification of potential transformation products formed during the proposed integrated technology (UASB—CWs), while ensuring environmental safety by assessing potential toxicity as well.

## Figures and Tables

**Table 1:** Influent wastewater conventional pollutants characteristics during different operational periods (average ± SD).

	Period	1st (April)	2nd (May)	3rd (December)
Parameter				
Samples (*n*)		8	9	8
EC (μS/cm)		1303 ± 124	1434 ± 73	1370 ± 274
Turb (NTU)		230 ± 63	309 ± 72	214 ± 118
pH		7.8 ± 0.1	7.7 ± 0.1	7.4 ± 0.2
TSS (mg/L)		329 ± 107	407 ± 105	302 ± 122
VSS (mg/L)		279 ± 89	355 ± 79	235 ± 111
BOD_5_ (mg/L)		308 ± 70	353 ± 47	447 ± n/a
tCOD (mg/L)		621 ± 179	748 ± 248	666 ± 274
sCOD (mg/L)		124 ± 59	133 ± 32	172 ± 85
NH_4_-N (mg/L)		51.1 ± 10.0	65.3 ± 9.7	60.7 ± 1.4
NO_3_-N (mg/L)		n.d. ^1^	n.d.	n.d.
TP (mg/L)		8.5 ± 2.5	11.1 ± 2.4	6.6 ± n/a
PO_4_-P (mg/L)		5.8 ± 1.1	7.4 ± 0.8	5.6 ± 1.7

^1^ not detected.

**Table 2:** Pilot system operational parameters during the sampling campaign periods (average ± SD).

	Period	1st (April)	2nd (May)	3rd (December)
Parameter				
Τ (°C)		15.9 ± 1.4	19.3 ± 1.7	16.8 ± 0.7
Q_pilot_ (m^3^/d)		41.2 ± 3.6	59.2 ± 0.2	76.1 ± 6.6
HRT_UASB_ (h)		24.0 ± 1.8	16.6 ± 0.1	12.8 ± 1.0
OLR_UASB_ (kg COD/m^3^-d)		0.6 ± 0.2	1.1 ± 0.4	1.2 ± 0.5
HRT_VSSF SAT CW_ (d)		3.1 ± 0.2	2.1 ± 0	1.7 ± 0.2
SLR_VSSF SAT CW_ (g TSS/m^2^-d)		25 ± 13	48 ± 10	34 ± 23
OLR_VSSF SAT CW_ (g COD/m^2^-d)		51 ± 20	98 ± 13	74 ± 29
Resting period_VSSF UNSAT CW_ (h)		7.8 ± 0.7	5.3 ± 0	4 ± 0.4
SLR_VSSF UNSAT CW_ (g TSS/m^2^-d)		0.8 ± 0.4	2.7 ± 1	4.6 ± 3.6
OLR_VSSF UNSAT CW_ (g COD/m^2^-d)		7.4 ± 2.3	12.6 ± 1.5	16.5 ± 5.3
NLR_VSSF UNSAT CW_ (g NH₄-N/m^2^-d)		3.7 ± 0.9	6.8 ± 0.7	7.4 ± 1.1
Q_conv-WWTP_ (m^3^/d)		63.4 ± 5.8	25.1 ± 4.2	10.5 ± 3.2

**Table 3:** Influent measured concentrations (ng/L) of the target CECs (average, standard deviation, min, max), frequency of detection for all sampling periods (*n* = 9 samples) and average daily mass loads (mg/d/1000 inh) of NSAIDs in Antissa, Lesvos, Greece.

Compound	Average	SD	Min	Max	Frequency of Detection	Average Mass Loads
Unit	(ng/L)				(%)	(mg/d/1000 inh)
IBU	6595	1841	4558	9296	100	1055
NPX	5267	1325	2734	6813	100	843
TCS	444	166	281	780	100	-
BPA	652	125	498	809	100	-
DCF	5462	1840	3225	7697	100	874
KFN	745	523	282	2067	100	119
5TTR	3249	1558	1127	5830	100	-
CBTR	8608	7504	2675	23,085	100	-
XTR	5681	4726	2010	13,290	100	-
OH-BTH	7392	6283	n.d.	16,698	67	-

## Data Availability

Dataset available on request from the authors.

## Supplementary Materials

The following supporting information can be downloaded at: https://www.mdpi.com/article/10.3390/molecules30132671/s1, Table S1: Average influent concentrations (ng/L) of the target CECs for each sampling period (n = 3) and for all periods (n = 9), Table S2: Average influent mass loads (mg/d) for each sampling period (n = 3) and for all periods (n = 9) in pilot system (UASB-SAT-UNSAT A), Table S3: Comparison of the total removal efficiency of pilot system (UASB-SAT-UNSAT A) and conventional WWTP for each period and each compound though ANOVA. Level of significance was set to 95%, Table S4: Data used for risk assessment, Table S5: Risk quotient (RQ) for the treated wastewater of the pilot system (UASB-SAT-UNSAT A), Table S6: Risk quotient (RQ) for the treated wastewater of the conventional WWTP. References [23,71,72,73] are cited in the Supplementary Materials.

## Abbreviations

The following abbreviations are used frequently throughout this manuscript:

UASB	Upflow anaerobic sludge blanket reactor
VSSF	Vertical subsurface flow
CW	Constructed wetland
CECs	Contaminants of emerging concern
EDCs	Endocrine disrupting chemicals
SAT	Saturated (vertical subsurface flow constructed wetland)
UNSAT	Unsaturated (vertical subsurface flow constructed wetland)
Conv-WWTP	Conventional wastewater treatment plant
BTRs	Benzotriazoles
XTR	Xylytriazole
CBTR	5-chlorobenzotriazole
5TTR	5-methyl-1H benzotriazole
BTH	Benzothiazole
OH-BTH	2 hydroxybenzothiazole
TCS	Triclosan
BPA	Bisphenol A
NSAIDs	Non-steroidal anti-inflammatory drugs
IBU	Ibuprofen
NPX	Naproxen
DCF	Diclofenac
KFN	Ketoprofen
RQ	Risk Quotient
