# Supplementary material for: The Fate of Contaminants of Emerging Concern in an Upflow Anaerobic Sludge Blanket Reactor Coupled with Constructed Wetlands for Decentralized Domestic Wastewater Treatment
Source: Molecules. 2025 Jun 20;30(13):2671. doi: 10.3390/molecules30132671 (PMC12251067; doi:10.3390/molecules30132671)
Supplement: Supplementary file 1 [file molecules-30-02671-s001.zip › molecules-3688541-supplementary.pdf]

Table S1. Average influent concentrations (ng/L) of the target CECs for each sampling period (n = 3) and for all periods (n = 9).

Compound	1st period (n = 3)	2nd period (n = 3)	3rd period (n = 3)	All periods (n = 9)
IBU	5,341±1,173	8,115±1,638	6,328±1,888	6,595±1,841
NPX	6,010±740	6,057±504	3,735±968	5,267±1,325
DCF	4,307±1,664	7,328±370	4,751±1,632	5,462±1,840
KFN	396±100	673±122	1,168±782	745±523
BPA	762±68	632±160	562±41	652±125
TCS	338±52	494±149	501±244	444±166
5TTR	3,277±589	2,302±1,881	4,167±1,791	3,249±1,558
CBTR	2,817±217	5,040±2,176	17,967±4,432	8,608±7,504
XTR	2,659±172	2,541±478	11,843±1,907	5,681±4,726
OH-BTH	8,154±1,391	14,021±2,695	ND	7,392±6,284

Table S2. Average influent mass loads (mg/d) for each sampling period (n = 3) and for all periods (n = 9) in pilot system (UASB-SAT-UNSAT A).

Compound	1st period (n = 3)	2nd period (n = 3)	3rd period (n = 3)	All periods (n = 9)
IBU	220±48	480±97	482±144	394±158
NPX	248±30	359±30	284±74	297±65
DCF	177±69	434±22	362±124	324±135
KFN	16±4	40±7	89±59	48±44
BPA	31±3	37±9	43±3	37±7
TCS	14±2	29±9	38±19	27±15
5TTR	135±24	136±111	317±136	196±127
CBTR	116±9	298±129	1367±337	594±613
XTR	110±7	150±28	901±145	387±393
OH-BTH	336±57	830±160	ND	389±371

Table S3. Comparison of the total removal efficiency of pilot system (UASB-SAT-UNSAT A) and conventional WWTP for each period and each compound though ANOVA. Level of significance was set to 95%.

	**1st**	**2nd**	**3rd**
**IBU**	WWTP ↓– PILOT	WWTP ↓– PILOT	-
**NPX**	WWTP ↓– PILOT	WWTP ↓– PILOT	-
**TCS**	-	WWTP ↓– PILOT	-
**BPA**	WWTP ↓– PILOT	WWTP ↓– PILOT	WWTP ↓– PILOT
**DCF**	WWTP ↓– PILOT	WWTP ↓– PILOT	-
**KFN**	-	WWTP ↓– PILOT	-
**5TTR**	WWTP ↓– PILOT	WWTP ↓– PILOT	-
**CBTR**	-	-	WWTP– PILOT ↓
**XTR**	-	-	-
**OH-BTH**	-	-	N.D.

Table S4. Data used for risk assessment.

Compound	Reference	EC_{50}/LC_{50} (mg L^{-1})		
		**Fish**	**Daphnia magna**	**Algae**
Diclofenac	Table S5, [1]	5.3	22.0	14.5
Ibuprofen	Table S5, [1]	42.0	9.0	4.0
Ketoprofen	Table S5, [1]	264.1	64.0	179.5
Naproxen	Table S5, [1]	193.3	166.3	625.5
Bisphenol A	Table S5, [1]	0.158	3.9	1.0
Triclosan	Table S5, [1]	0.260	0.390	0.0014
5,6-dimethyl-1H-benzotriazole (xylytriazole)	Table S5, [1]	9.4	19.3	2.5
2-hydroxybenzothiazole	Table S5, [1]	3.8	15.1	0.611
5-chlorobenzotriazole	Fish: [2]<br>D. Magna: [3]<br>Algae: [4]	19.0	28.7	38.7
5-methyl-1H-benzotriazole	Fish, D. Magna: [3]<br>Algae: [4]	22.0	50.9	22.0

Table S5. Risk quotient (RQ) for the treated wastewater of the pilot system (UASB-SAT-UNSAT A).

**Compound**	**Worst-case scenario**			**Base-case scenario**		
	**Fish**	***Daphnia magna***	**Algae**	**Fish**	***Daphnia magna***	**Algae**
IBU	0.03	0.13	0.30	0.02	0.09	0.20
NPX	0.01	0.01	0.00	0.01	0.01	0.00
DCF	0.54	0.13	0.20	0.22	0.05	0.08
KFN	0.00	0.01	0.00	0.00	0.00	0.00
BPA	**0.87**	0.04	0.14	**0.53**	0.02	0.08
TCS	0.79	**0.52**	**145.86**	0.28	**0.19**	**52.81**
5TTR	0.11	0.05	0.11	0.04	0.02	0.04
CBTR	0.19	0.13	0.09	0.09	0.06	0.04
XTR	0.19	0.09	0.71	0.07	0.03	0.26
OH-BTH	0.26	0.06	1.59	0.03	0.01	0.18
**Cumulative RQ**	2.99	1.17	149.01	1.29	0.48	53.70

Table S6. Risk quotient (RQ) for the treated wastewater of the conventional WWTP.

**Compound**	**Worst-case scenario**			**Base-case scenario**		
	**Fish**	**Daphnia Magna**	**Algae**	**Fish**	**Daphnia Magna**	**Algae**
IBU	0.20	0.92	2.08	0.09	0.40	0.91
NPX	0.03	0.03	0.01	0.02	0.02	0.00
DCF	1.45	0.35	0.53	0.74	0.18	0.27
KFN	0.00	0.01	0.00	0.00	0.01	0.00
BPA	**5.02**	0.20	0.79	**2.93**	0.12	0.46
TCS	1.51	**1.01**	**280.45**	0.71	**0.47**	**132.18**
5TTR	0.05	0.02	0.05	0.01	0.00	0.01
CBTR	0.21	0.14	0.10	0.09	0.06	0.04
XTR	0.14	0.07	0.52	0.06	0.03	0.21
OH-BTH	1.01	0.25	6.28	0.21	0.05	1.28
**Cumulative RQ**	9.62	3.00	290.82	4.85	1.34	135.38

References

1. Thomaidi, V.S.; Stasinakis, A.S.; Borova, V.L.; Thomaidis, N.S. Is There a Risk for the Aquatic Environment Due to the Existence of Emerging Organic Contaminants in Treated Domestic Wastewater? Greece as a Case-Study. *Journal of Hazardous Materials* **2015**, *283*, 740–747, doi:10.1016/j.jhazmat.2014.10.023.
2. Sampaio, C.F.; Gravato, C.; De Oliveira, D.P.; Dorta, D.J. Deleterious Effects of Benzotriazoles on Zebrafish Development and Neurotransmission: 5-Chloro-Benzotriazole versus 1H-Benzotriazole. *Science of The Total Environment* **2024**, *912*, 168741, doi:10.1016/j.scitotenv.2023.168741.
3. Giraudo, M.; Douville, M.; Cottin, G.; Houde, M. Transcriptomic, Cellular and Life-History Responses of Daphnia Magna Chronically Exposed to Benzotriazoles: Endocrine-Disrupting Potential and Molting Effects. *PLoS ONE* **2017**, *12*, e0171763, doi:10.1371/journal.pone.0171763.
4. Gatidou, G.; Anastopoulou, P.; Aloupi, M.; Stasinakis, A.S. Growth Inhibition and Fate of Benzotriazoles in Chlorella Sorokiniana Cultures. *Science of The Total Environment* **2019**, *663*, 580–586, doi:10.1016/j.scitotenv.2019.01.384.
